# Digenic Inheritance in Cystinuria Mouse Model

**DOI:** 10.1371/journal.pone.0137277

**Published:** 2015-09-11

**Authors:** Meritxell Espino, Mariona Font-Llitjós, Clara Vilches, Eduardo Salido, Esther Prat, Miguel López de Heredia, Manuel Palacín, Virginia Nunes

**Affiliations:** 1 Molecular Genetics Laboratory, Bellvitge Biomedical Research Institute (IDIBELL), Hospitalet de Llobregat, Barcelona, Spain; 2 Center for Biomedical Network Research on Rare Diseases (CIBERER), U730, Hospitalet de Llobregat, Barcelona, Spain; 3 Pathology Department, Canarias University Hospital La Laguna, Tenerife, Spain; 4 Center for Biomedical Network Research on Rare Diseases (CIBERER), U740, La Laguna, Tenerife, Spain; 5 Institute of Research in Biomedicine (IRB), Barcelona, Spain; 6 Center for Biomedical Network Research on Rare Diseases (CIBERER), U731, Barcelona, Spain; 7 Biochemistry and Molecular Biology Department, Barcelona University (UB)), Barcelona, Spain; 8 Genetic Section, Physiology Science II Department, Barcelona University (UB), Barcelona, Spain; IGBMC/ICS, FRANCE

## Abstract

Cystinuria is an aminoaciduria caused by mutations in the genes that encode the two subunits of the amino acid transport system b^0,+^, responsible for the renal reabsorption of cystine and dibasic amino acids. The clinical symptoms of cystinuria relate to nephrolithiasis, due to the precipitation of cystine in urine. Mutations in *SLC3A1*, which codes for the heavy subunit rBAT, cause cystinuria type A, whereas mutations in *SLC7A9*, which encodes the light subunit b^0,+^AT, cause cystinuria type B. By crossing *Slc3a1* ^*-/-*^ with *Slc7a9* ^-/-^ mice we generated a type AB cystinuria mouse model to test digenic inheritance of cystinuria. The 9 genotypes obtained have been analyzed at early (2- and 5-months) and late stage (8-months) of the disease. Monitoring the lithiasic phenotype by X-ray, urine amino acid content analysis and protein expression studies have shown that double heterozygous mice (*Slc7a9* ^+/-^ *Slc3a1* ^+/-^) present lower expression of system b^0,+^ and higher hyperexcretion of cystine than single heterozygotes (*Slc7a9* ^+/-^ *Slc3a1* ^+/+^ and *Slc7a9* ^+/+^ *Slc3a1* ^+/-^) and give rise to lithiasis in 4% of the mice, demonstrating that cystinuria has a digenic inheritance in this mouse model. Moreover in this study it has been demonstrated a genotype/phenotype correlation in type AB cystinuria mouse model providing new insights for further molecular and genetic studies of cystinuria patients.

## Introduction

Cystinuria is an inherited aminoaciduria caused by mutations in *SLC7A9* and *SLC3A1* that encode for the two subunits b^0,+^AT and rBAT, respectively. Intracellular assembly of both subunits by a disulfide bridge form a heterodimer that is expressed in the apical membrane of epithelial cells and is required for functional amino acid transport of system b^0,+^ [1]. The rBAT/b^0,+^AT heterodimer mediates Na^+^‐independent exchange of external cationic amino acids and cystine with neutral amino acids [2]. This complex is responsible for >90% of cystine reabsorption in epithelial cells of the renal proximal tubule and small intestine [3,4]. Thus, system b^0,+^ malfunction causes an inadequate reabsorption of cystine and dibasic amino acids (lysine, arginine and ornithine) triggering their urine hyperexcretion, the hallmark of cystinuria [5]. The clinical symptoms of cystinuria are caused by cystine’s precipitation due to is poor solubility, forming stones in the urinary tract that cause obstruction, infection and, ultimately, chronic kidney disease in patients [6].

Cystinuria caused by mutations in *SLC3A1* (rBAT) has an autosomal recessive inheritance pattern (OMIM 600918) and mutations in *SLC7A9* (b^0,+^AT) follows a partially autosomal dominant inheritance pattern (OMIM 220100). The lack of a genotype/phenotype correlation has led to propose a genetic classification for cystinuria defined as type A if mutations are found in *SLC3A1*, type B if mutations are found in *SLC7A9* and type AB when one mutation is found in each gene [7]. To date, 1.6% of cystinuria patients with lithiasic phenotype have been classified as type AB. In fact, all of these cases are carriers of three mutations implying a triallelic genotype; that is, AAB and BBA types [8]. Obligate heterozygous type AB carriers were only detected in 3 cystinuric patients’ relatives, showing mild cystine hyperexcretion (2 to 3-fold higher than normal) although far below the range found in patients (>20-fold). In addition these individuals have never shown cystine urolithiasis [9–11], thus they have not been classified as cystinuria patients [9–11]. As obligate type AB (double heterozygous) individuals who develop cystinuria have not been found, digenic inheritance of cystinuria was ruled out.

To date, nearly 600 mutated alleles in *SLC3A1* and over 400 in *SLC7A9* have been reported in patients from different countries [8]. Although 1–2% of all cases of renal lithiasis and 6–8% of pediatric renal lithiasis were diagnosed as cystinuria patients [12], 13% of analyzed alleles of cystinuria patients remain to be identified [8]. Mutations in non-explored regions of *SLC3A1* and *SLC7A9* could be responsible for the cystinuria phenotype in these unidentified cases. Other possibility is that partial loss of function of both genes that cause cystinuria (i.e. polymorphisms, non-detected mutations or combination of both) might explain the remaining cases with cystinuria phenotype. Mutations in other genes involved in cystinuria are unlikely because, to our knowledge, genetic linkage to the two-cystinuria loci has not been excluded in any of the genetically unexplained patients (M.Font-LLitjòs, M.Palacín and V.Nunes; unpublished results).

Three mouse models of cystinuria have been described showing a clinical and biochemical phenotype rather close to humans. Mutations in rBAT leading to Asp140Gly [13] or Gly1232Ala [14] exhibited human type A cystinuria phenotype, and disruption of exon 3 to 9 of b^0,+^AT gene generates the features of human type B cystinuria [15]. By crossing both, type A (Asp140Gly) and B (disruption of exon 3 to 9) mouse models, double heterozygous mice (*Slc3a1* ^+/-^ *Slc7a9* ^+/-^) have been generated to determine if partial loss of function of one of those proteins and digenic inheritance of cystinuria-related genes could induce urolithiasis in mice.

## Results

### Viability of cystinuria type AB mice model

In order to unravel if cystinuria has a digenic inheritance, a second-generation breeding in mixed background C3HeB/C57Bl6 cystinuria type AB mice model has been generated. After obtaining 1152 mice, frequency of all 9 genotypes follows a normal pattern of Mendel's segregation (Table 1) demonstrating that the absence of both subunits, which form system b^0,+^ and causes cystinuria, are not lethal in this murine model. Body weight of either double mutant (*Slc7a9* ^-/-^ *Slc3a1* ^-/-^) nor double heterozygous mice (*Slc7a9* ^+/-^ *Slc3a1* ^+/-^) does not show differences compared to both single mutant cystinuria mice models (*Slc7a9* ^-/-^ *Slc3a1* ^+/+^ and *Slc7a9* ^+/+^ *Slc3a1* ^-/-^) (S1A Fig) and with wild type group (S1B Fig), respectively. Thus, *a priori*, the absence of any of the subunits of the transport system b^0,+^ does not affect either viability nor body weight in these animals.

**Table 1:** Inheritance percentage of F2 generation.

*Slc7a9*	*Slc3a1*	Male	Observed	Female	Observed	Expected
+/+	+/+	48	7,6	42	7,9	6,3
+/+	+/-	121	19,3	109	20,5	12,5
+/+	-/-	35	5,6	38	7,1	6,3
+/-	+/+	94	15	86	16,2	12,5
+/-	+/-	138	22	122	22,9	25
+/-	-/-	62	9,9	48	9,0	12,5
-/-	+/+	41	6,5	30	5,6	6,3
-/-	+/-	57	9,1	41	7,7	12,5
-/-	-/-	24	3,8	16	3,0	6,1
	TOTAL	620		532		

Data from 1152 offspring obtained from 10 different couples of F2 double heterozygous progenitors. Male/Female: Number of animals of each genotype. Observed: Percentage of newborn for each genotype. Expected: Percentage of segregation according to Mendel inheritance. No differences were found analyzing data with Chi-square test (p-value = 0,98 comparing real and expected frequency).

### Cystinuria phenotype characterization

**Fig 1:**
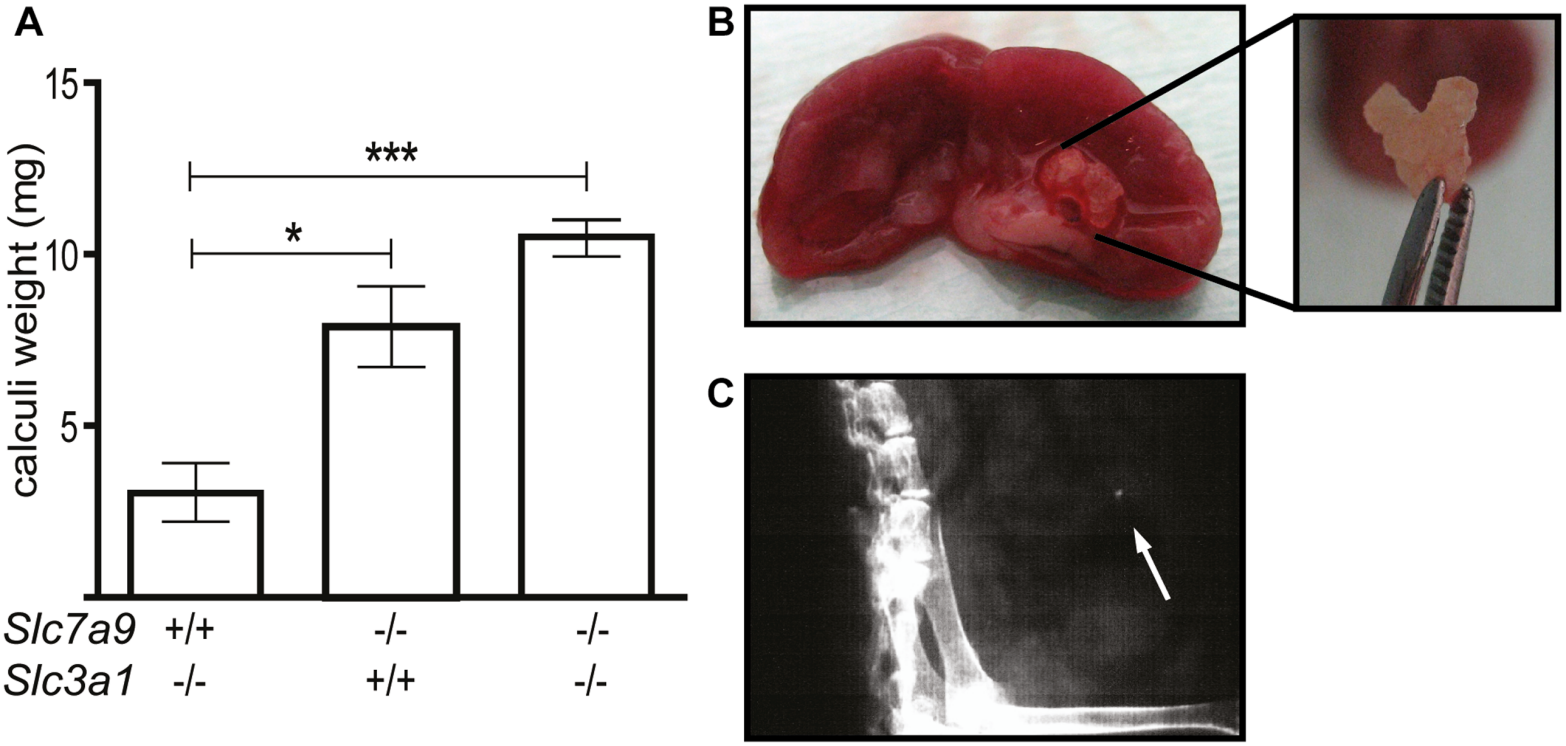
Characterization of lithiasic phenotype. **A**. Graph shows kidney calculi weight. Mean ± S.E.M of calculi weight (mg) of single mutants (*Slc7a9* ^*-/-*^ *Slc3a1* ^*+/+*^ and *Slc7a9* ^+/+^ *Slc3a1* ^*-/-*^) and double mutant (*Slc7a9* ^-/-^ *Slc3a1* ^*-/-*^) are represented. 5 to 7 calculi of male mice at 5 months of age were weighted for each genotype. Student’s t-test is represented as * p‐<0,05 and ***p<0,001. **B**. Example of a staghorn calculi found in double mutant that fills the whole medulla. **C**. Example of X-ray from a double heterozygous mouse (*Slc7a9* ^+/-^ *Slc3a1* ^*+/-*^) where the balder stone is indicated with an arrow.

Calculi formation has been analyzed by X-ray at 2, 5 and 8 months of age. Surprisingly, 5 out of 138 double heterozygous mice (*Slc7a9* ^+/-^ *Slc3a1* ^+/-^), which represent 4% of the animals, show calculi formation. Despite of this low percentage of lithiasis, this result demonstrates that partial loss of both subunits of system b^0+^ can cause a lithiasic phenotype (Table 2). Indeed, analysis of calculi formation has shown that double mutants (*Slc7a9* ^*-/-*^ *Slc3a1* ^*-/-*^) have both higher lithiasis prevalence (Table 2) and higher stone size (Fig 1A), than single mutants (*Slc7a9* ^‐/‐^ *Slc3a1* ^*+/+*^ and *Slc7a9* ^+/+^ *Slc3a1* ^*-/-*^). All double mutants presented calculi formation at 8 months of age and almost 50% of them presented a severe phenotype exhibiting calculi in both kidneys and bladder at the same time (Table 2). Concomitantly with the most severe lithiasic phenotype of the double mutant, kidney stones from animals with this genotype revealed a typical shape that has not been found yet in the other cystinuria mouse models. 12% of double mutants (3 of 24 *Slc7a9* ^-/-^ *Slc3a1* ^-/-^ mice) showed renal calculi that filled the cavity of the renal papilla acquiring a staghorn shape, which collapsed the kidney and led to renal failure (Fig 1B).

**Table 2:** Percentage of calculi formation and lithiasis prevalence.

Genotype		Bladder			Kidney			% Lithiasis			Bladder and kidney			% Lithiasis in bladder and both kidneys		
*Slc7a9*	*Slc3a1*	2m	5m	8m	2m	5m	8m	2m	5m	8m	2m	5m	8m	2m	5m	8m
**+/-**	**+/-**	4/90	4/90	4/90	0/90	0/90	0/90	4	4	4	0/90	0/90	0/90	0	0	0
**+/+**	**-/-**	7/24	12/22	10/13	6/24	10/22	5/13	54	83	92	0/24	4/22	3/13	0	18	25
**-/-**	**+/+**	9/22	15/21	19/21	7/22	8/21	6/20	73	91	91	0/22	4/20	6/20	0	20	30
**-/-**	**-/-**	7/22	16/22	8/13	5/22	5/20	11/13	55	73	100	0/22	5/20	6/13	0	25	46

Table shows the results of tracking the calculi formation by X-ray. Number of mice that have developed calculi in bladder, kidney or in both organs (Bladder and kidney) at 2, 5 and 8 months of age (2m, 5m and 8m) are indicated (mice with calculi / mice studied). Percentages of mice with calculi (% Lithiasis) and with simultaneous calculi formation (% Lithiasis in bladder and both kidneys) are indicated.

**Fig 2:**
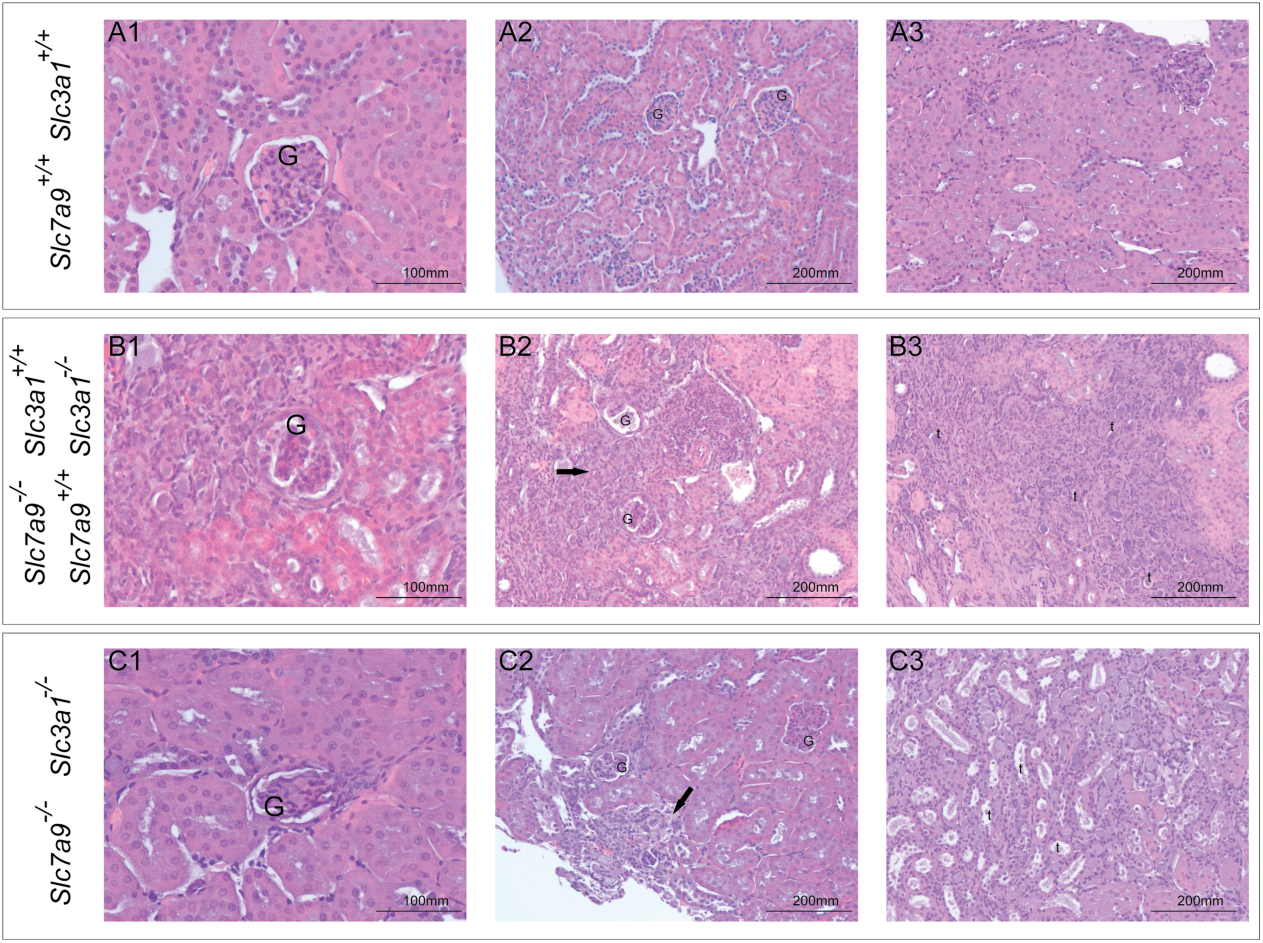
Histopathological studies of renal sections. Hematoxylin/eosine staining. Analysis were done in wild‐type mice (row A), single mutants (row B) and double mutant (row C) mice. In B and C groups; only kidneys with calculi were analyzed. Column 1 shows glomerulus (G). **A1**: normal glomerulus; **B1**: glomerulus with partial collapse of capillary loops; **C1**: glomerulus with global matrix increase. Column 2 shows interstitial inflammation (arrow) and tubular atrophy. **A2**: normal tissue; **B2**: high level of interstitial inflammation; **C2**: interstitial inflammation and tubular atrophy. Column 3 shows renal tubules (t). **A3**: normal renal cortex; **B3**: tubular atrophy in renal cortex; **C3**: tubular atrophy with lumen dilatation.

Hematoxylin/eosin stained sections of kidneys from the 9 genotypes were analyzed (Fig 2). Necropsy and histopathology reveal that lithiasic kidney develops renal damage as glomerulosclerosis, renal tubules dilatation and focal interstitial inflammation in different degrees. Histological results correlate to different parameters as permanence, quantity or size of the calculi rather than to the genotype. Thereby, double homozygous mutants, which have more severe lithiasic phenotype, present strongest renal damage (Fig 2 row C) comparing with single homozygous mutants (Fig 2 row B). Single heterozygous mice, wild type and double heterozygous mice, even with lithiasic phenotype; do not show kidney damage (Fig 2 row A). This observation could be explained by the small size of the calculi present in the double heterozygous mutants, which could be detected by X-ray but impossible to extract during the necropsy because, due to its small size, have been impossible to localize. Furthermore, in double heterozygotes, stones have only been detected in bladder, never in kidneys (Fig 1C).

### Urine hyperexcretion

**Fig 3:**
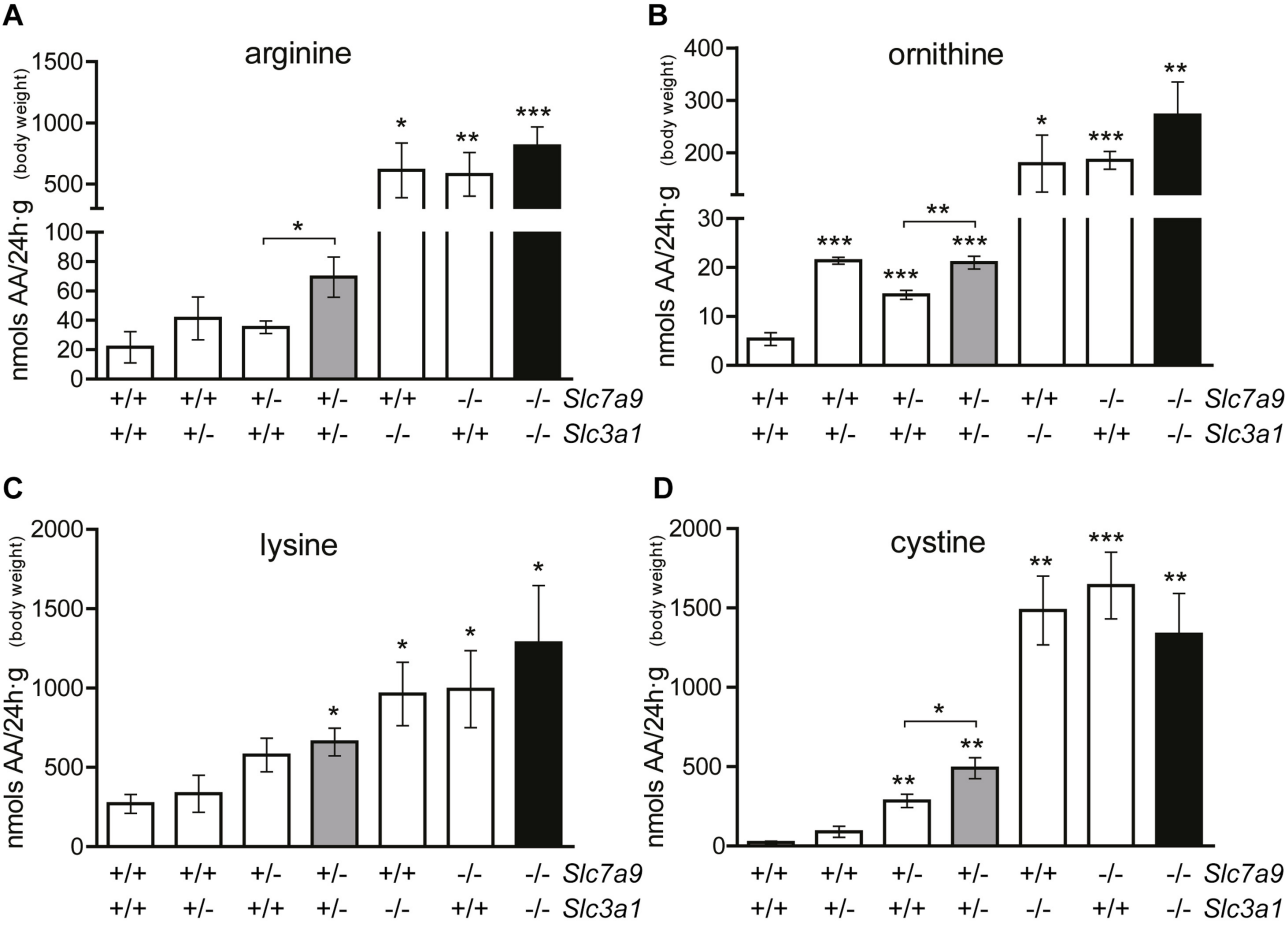
Urine amino acid content. Mean±S.E.M. of urine amino acids (nmols amino acid/24h∙g) of arginine (**A**), ornithine (**B**), lysine (**C**) and cystine (**D**) from 3 months‐old male mice (n≥7 animals for each genotype) are represented. Student’s t-test is represented as * p<0,05; **p<0,01 and ***p<0,001. Bars which represent double heterozygous (*Slc7a9* ^*+/-*^ *Slc3a1* ^*+/-*^) and double mutants (*Slc7a9* ^‐-/-^ *Slc3a1* ^-/-^) are filled in gray and black, respectively.

In order to determine the function of system b^0,+^ amino acids content of 24h urine samples have been analyzed. Double heterozygous mice, which showed calculi formation in 4% of the cases, show slight differences in dibasic amino acids urine excretion compared with singles heterozygotes animals (Fig 3A, 3B and 3C) and hyperexcrete 5- and 2-fold cystine in urine compared with single heterozygous mice (Fig 3D). *Slc7a9* heterozygous mice are classified as non‐type I cystinuria which hyperexcretes cystine and dibasic amino acids but does not show lithiasic phenotype [15]. Thus, double levels of cystine in urine could reach its solubilization threshold [16], triggering calculi formation and explaining the 4% of lithiasis found in double heterozygous mice (Table 2).

**Fig 4:**
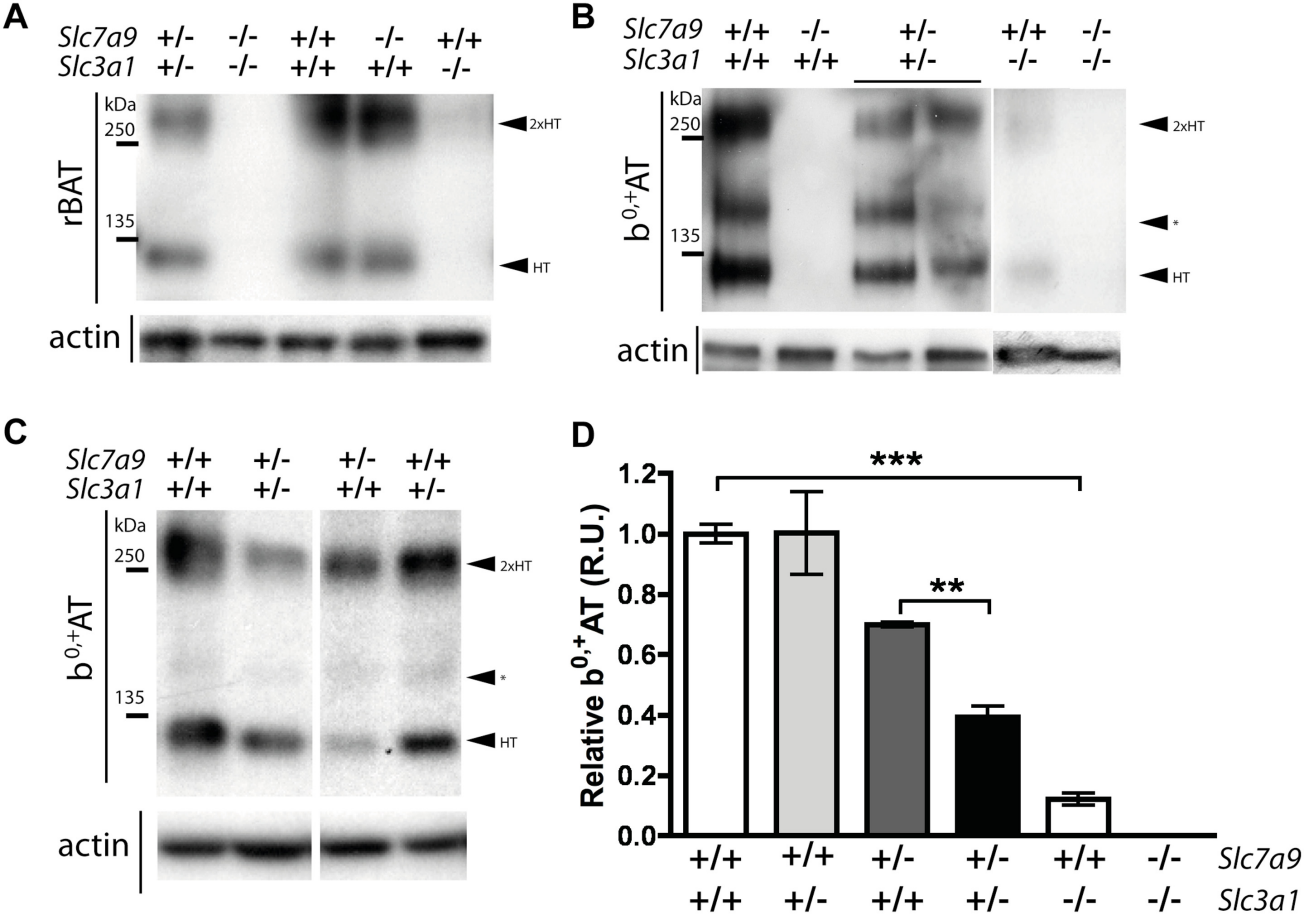
Western blot of brush‐border kidney membranes. Protein analysis of rBAT and b^0,+^AT in kidney brush‐border membranes of different genotypes. Fifty micrograms of protein were loaded in all lanes and run in non‐reducing conditions in 7% acrylamide SDS-page. Molecular mass standard (kDa) are indicated. **A.** Antibodies against rBAT and β-actin were used and are indicated on the left side. Arrows from right side indicate the heterodimer (HT) and, as well the oligomeric structure of 2 heterodimers (2xHT). Wild‐type (*Slc7a9* ^+/+^ *Slc3a1* ^+/+^), single mutants (*Slc7a9* ^+/+^ *Slc3a1* ^-/-^ and *Slc7a9* ^-/-^ *Slc3a1* ^+/+^), double mutant (*Slc7a9* ^-/-^ *Slc3a1* ^-/-^) and double heterozygous (*Slc7a9* ^+/-^ *Slc3a1* ^+/-^) are shown. **B and C.** Antibodies against b^0,+^AT and β-actin were used and are indicated on the left side. Arrows from right side indicate the rBAT/b^0,+^AT heterodimer (HT), the oligomeric structure of 2 heterodimers (2xHT) and a band corresponding to a complex, variable among experiments, formed by the oligomerization of b^0,+^AT detached from rBAT artifact of brush-border extraction and/or SDS-page conditions (*). (B) Wild type, single mutants, double mutant and double heterozygous mice are represented. (C) Wild type, double and both single heterozygous (*Slc7a9* ^+/-^ *Slc3a1* ^+/+^ and *Slc7a9* ^+/+^ *Slc3a1* ^+/-^) samples are shown. **D**. Graph representing the densitometry analysis of b^0,+^AT corrected by β-actin of double and single heterozygous, rBAT mutant and double mutant samples compared to wild type. The mean of 3–4 independent western blot analysis is represented.

Surprisingly, double homozygous mutants (*Slc7a9* ^*-/-*^ *Slc3a1* ^*-/-*^) show a slight decrease in cystine hyperexcretion compared to both single homozygous mutants (Fig 4A). In the presence of calculi, cystine is forming the stone(s) and there is less free cystine in urine. As lithiasis prevalence and size of stones in double homozygous mutants are higher than in both single homozygous mice (Table 2), a significant part of the cystine might be forming the calculi. Therefore, it is feasible that levels of cystine in urine show a tendency to decrease in the double mutants compared to single mutants.

### b^0,+^AT/ rBAT protein analysis

Western blot analysis of apical renal membranes was performed to test the presence/absence of b^0,+^AT (*Slc7a9*) and rBAT (*Slc3a1*) subunits and confirm the correlation of amount of protein *versus* genotypes. Double homozygous mutant (*Slc7a9* ^-/-^ *Slc3a1* ^-/-^) did not show either b^0,+^AT nor rBAT demonstrating that double mutant mice completely abolish the presence of both subunits of system b^0,+^ (Fig 4A and 4B). Protein analysis also shows 10% of remaining rBAT/b^0,+^AT heterodimer in renal brush-borders of Asp140Gly rBAT homozygous mutant (*Slc7a9* ^+/+^ *Slc3a1* ^-/-^) (Fig 4A, 4B and 4D). As rBAT mutant mouse model used in this study has a point mutation not being a total knockout, this result demonstrates that some mutated rBAT reaches the apical membrane conferring some system b^0,+^ activity to the renal cell. In contrast, ablation of b^0,+^AT (*Slc7a9* ^-/-^ *Slc3a1* ^+/+^) completely abolished the expression of the heterodimer (Fig 4B). Additionally, the absence of b^0,+^AT shows the presence of another rBAT-associated subunit forming a 120 kDa heterodimer (Fig 4A). Western blot of apical membranes in non-reducing conditions of *Slc7a9* homozygous knockout confirms, as co-immunopurification studies described before [17], that all b^0,+^AT present in renal brush-border heterodimerizes with rBAT, but not all rBAT forms heterodimers with b^0,+^AT.

Since rBAT antibody in non-reducing conditions detects both heterodimers, rBAT/b^0,+^AT and rBAT/unknown light subunit, to assess the correct quantification of system b^0,+^ brush-border samples were analyzed using b^0,+^AT antibody (Fig 4B and 4C). This analysis shows a lower presence of the heterodimer (rBAT/b^0,+^AT) in renal apical membranes of the double heterozygous mice (*Slc7a9* ^+/-^ *Slc3a1* ^+/-^) comparing with both single heterozygous mutants (*Slc7a9* ^+/+^ *Slc3a1* ^+/-^ and *Slc7a9* ^+/-^ *Slc3a1* ^+/+^) (Fig 4C and 4D). Densitometric analysis shows that the presence of system b^0,+^ in renal apical membranes of double heterozygous mice is around 40% lower than b^0,+^AT heterozygous mice (Fig 4D). Double heterozygous mice show lithiasis in 4% of the mice at 8 months of age (Table 2) and higher cystine hyperexcretion than both single heterozygous mice (Fig 3D), indicating a correlation between levels of rBAT/b^0,+^AT heterodimer in brush-borders with both hyperexcretion and lithiasic phenotypes. Thus, lower quantity of the rBAT/b^0,+^AT heterodimer entails higher levels of cystine in urine indicating a system b^0,+^ malfunction, and eventually cystine lithiasis.

## Discussion

The scarce number of patients classified as obligate cystinuria type AB and the high percentage (13%) of cystinuria patients that remain to be classified encouraged us to generate a cystinuria type AB *in vivo* model to analyze genotype/phenotype correlation and test the existence of possible digenia in cystinuria. Tracking the lithiasic phenotype by X-ray allowed us to detect calculi formation in 4% of the double heterozygous mice (*Slc7a9* ^+/-^ *Slc3a1* ^+/-^) (Table 2) demonstrating that cystinuria has a digenic inheritance pattern in this mouse model. Concomitantly with the calculi formation, double heterozygous mice have higher concentration of cystine in urine (Fig 3D) besides lower amount of rBAT/b^0,+^AT heterodimer at the renal apical membrane (Fig 4C and 4D). These results are clear evidence that half-dose of both subunits of the transport system b^0,+^ decreases renal cystine reabsorption allowing the development of cystine urolithiasis. The low lithiasis prevalence, as shown in double heterozygous mice, could be the explanation why digenic inheritance pattern in cystinuria patients has not been detected yet. Nowadays, only 3 type AB cases have been identified. Therefore, finding a stone former patient would not be easy to detect, assuming a similar or lower percentage of lithiasis than the one observed in type AB mice (4%). In addition, double heterozygous calculi have shown a very small size (Fig 1C) and have only been found in mice bladders. So, due to the tiny size of the calculi and the low prevalence, digenic cystinuria carriers could escape to the screening not being diagnosed as cystinuria patients and/or misclassified as spontaneous urolithiasis cases if an urine cystine analysis content is not performed. These results hint that patients who are carriers of double heterozygous mutations could be classified as risk group to develop urolithiasis. Cystine hyperexcretion combined with external factors such as water, protein or salt intake could trigger cystine precipitation and lithiasic phenotype [8]. For this reason, we strongly suggest to alert and inform the digenic carriers to follow the cystinuria food and drink intake guideline to prevent the calculi manifestation [8].

Digenic inheritance of cystinuria in mice also could contribute to understand unclassified cystinuria patients. As it has been demonstrated, partial loss of function of system b^0,+^ ends up in full-blow cystinuria; and rBAT/b^0,+^AT heterodimer partial-dose could be caused by different factors such as mutations in non-codifying regions or due to polymorphisms. Therefore, deeply genetic study of unclassified cystinuria patients is recommended.

In humans, a cystinuria phenotype/genotype correlation was discarded because patients sharing the same mutations show marked lithiasic phenotype and cystine hyperexcretion differences [5,9]. Cystine crystals are formed when several conditions are met besides levels of hyperexcreted cystine. It has been reported that many different external factors such as dietary and lithiasis modulating genes all contribute to calculi formation [6]. Although all cystinuria type AB generated mice share same life-style (diet and environment) and genetic background, not all mice with same mutation formed calculi. Even so, prevalence and severity of lithiasic phenotype differs between genotypes. It has been observed that prevalence and severity of lithiasis is increased in the double homozygotes (*Slc7a* ^-/-^ *Slc3a1* ^-/-^) compared with the single ones (*Slc7a9* ^-/-^ *Slc3a1* ^+/+^ and *Slc7a9* ^+/+^ *Slc3a* ^-/-^) (Table 2 and Fig 1). This strongest lithiasic phenotype is in agreement with a recently reported patient which is carrier of 2 homozygous mutations, one in each subunit, that shows excessively cystine hyperexcretion and an aggravated lithiasic phenotype (very early calculi onset, within the first 14 month of age) [11]. In this line, protein analysis of system b^0,+^ (Fig 4) revealed some interesting findings: 1) rBAT mutant mice are not fully deficient in rBAT/b^0,+^AT heterodimer in renal apical membrane, 2) the existence of an unknown light subunit that heterodimerizes with rBAT and 3) double mutant mice (*Slc7a9* ^-/-^ *Slc3a1* ^-/-^) completely abolish the presence of b^0,+^ system and also the rBAT/unknown subunit heterodimer from renal apical membranes. There is no evidence of which could be the transport activity of rBAT/unknown subunit, although the fractional excretion of cystine in *Slc7a9* ^-/-^ mice (i.e., complete ablation of system b^0,+^) is only 11%, attributing the remaining cystine reabsorption to uncharacterized transport systems [18]. Considering that rBAT/unknown subunit could play a role in cystine apical transport, the double mutant mice would have less cystine renal reabsorption compared with both single mutants (*Slc7a9* ^-/-^ *Slc3a1* ^+/+^ and *Slc7a9* ^+/+^ *Slc3a1* ^-/-^). Although urine amino acid content does not show differences in cystine content between these genotypes (Fig 3D), because the main part is forming the calculi, differences in the lithiasis severity (Table 2), a tendency in the kidney calculi size (Fig 1A) and the presence of staghorn calculi (Fig 1B) have been detected. Thus, accordingly with our hypothesis, where rBAT/unknown subunit could participate in cystine reabsorption besides system b^0,+^, the most severe lithiasis phenotype has been found in double mutant mice, which completely abolish both heterodimers.

Genotype/phenotype correlation is also found when comparing homozygous b^0,+^AT knockout (*Slc7a9* ^-/-^ *Slc3a1* ^+/+^) and rBAT mutant (*Slc7a9* ^+/+^ *Slc3a1* ^-/-^) mice. b^0,+^AT knockout has lost completely system b^0,+^ transport activity and should have intact rBAT/unknown subunit heterodimer, whereas the rBAT mutant mice conserve approximately 10% of system b^0,+^ and apparently lacks the heterodimer rBAT/unknown subunit (i.e., as resembling the double mutant, which shows no presence of rBAT heterodimers in renal brush-borders (Fig 4A)). The fact that lithiasis is more severe in b^0,+^AT knockout than in the rBAT mutant mice strongly suggest that system b^0,+^ is more conspicuous for cystine reabsorption than the not yet defined transport activity of the rBAT/unknown subunit. Alternatively, we cannot exclude the possibility that genetic factors could contribute in lithiasis phenotype. In type B cystinuria mice (*Slc7a9* ^-/-^ *Slc3a1* ^+/+^) lithiasic phenotype increases from 43% of calculi formation at 2-months of age in the second filial generation to ~85% in the sixth filial generation obtained by intercrossing lithiasic mice in pure C57Bl/6 background (L.Feliubadaló, M.Palacín and V.Nunes; unpublished results). In fact, lithiasis percentage of mice with same mutation varies among mice strains, such as *Slc3a1* ^D140G^ mouse line in C3HeB pure background that shows 100% of calculi formation [13] whereas in a mixed C57Bl6/J-C3H background is 90% (Table 2); being another evidence that calculi formation could vary due to the genetic background itself. Thus, genetic factors might affect lithiasis differences between genotypes. As this study has been performed using a mixed genetic background (C57Bl6/J-C3H), genes contiguous with both mutations could be accidentally slanted in either (*Slc7a9* ^-/-^ *Slc3a1* ^+/+^) or (*Slc7a* ^+/+^ *Slc3a1* ^-/-^) mice. It means that genes adjacent to *Slc7a9* are manly in C57Bl6/J background in the b^0,+^ AT knockout mice meanwhile in rBAT mutant mice, genes bordering *Slc3a1* pertain to C3H strain. Lithiasis modulating genes responsible for cystine calculi formation are unknown and its identification using cystinuria mouse models is in progress.

Summarizing, generation and characterization of type AB cystinuria mouse model exhibited a digenic inheritance where 4% of double heterozygous mice (*Slc7a9* ^+/-^ *Slc3a1* ^+/-^) present lithiasis phenotype. Moreover, analysis of the heterodimer in renal apical membrane of double and single mutants provided more information of the molecular basis of cystinuria. In this study the impact of a genetic factor, in addition to the mutations in the transport system b^0,+^, that could modulate lithiasis prevalence and severity was confirmed. Indeed, results shown in this work suggest that rBAT/unknown subunit heterodimer could play a role in renal cystine reabsorption. Generation of the type AB cystinuria mice provide a powerful tool for identification of both lithiasis modifiers genes and the unknown light subunit that heterodimerizes with rBAT.

## Methods

All protocols used in this study were reviewed and approved by the Institutional Animal Care and Use Committee at IDIBELL in a facility accredited by the Association for the Assessment and Accreditation of Laboratory Animal Care International (AALAC) (B-9900010/3866). Mice procedures were designed and done according with the highest scientific, humane, and ethical principles.

### Mouse model

Cystinuria type A and B mouse models were inter-crossed to generate cystinuria type AB model. *Slc7a9* knockout mice in C57BL/6J pure background [15] were crossed with *Slc3a1* ^D140G^ mouse line in C3HeB pure background [13] obtaining a F1 generation in a mixed C57Bl6/J-C3H genetic background. Double heterozygous mice (*Slc7a9* ^+/-^ *Slc3a1* ^+/-^) were backcrossed obtaining 620 mice of F2 generation with the 9 expected genotypes. At the end of each procedure mice were euthanized in a CO_2_ chamber. To perform all experiments described in this study only male mice have been analyzed because previous work using cystinuria mouse models [13,15] evidenced that there is no reason to suspect that results will differ between genders.

### Genotyping

DNA extraction was performed from tail biopsy. Tail samples were incubated at 56°C ON with 0.4mg/mL of Proteinase K in lysis buffer (10mM Tris pH8.0, 100mMNaCl, 10mM EDTA pH8.0, 0.5% SDS). DNA was precipitated using isopropanol and resuspended in DNAse-free water. *Slc7a9* was genotyped as published in [15]. To genotype *Slc3a1* point mutation, PCR conditions were 98°C(3’) [98°C(25”), 58°C(2’), 72°C(3’)]x30C 72°C(1’) using both primers, For:CCGTGGTTTCCGTGTTCCTG and Rev:GCTTGCTGACTTCTGCTGCG. Product of rBAT PCR was purified with *QIAquick PCR Purification Kit* (Quiagen) prior to sequencing using Big Dye system (Life technologies) and reverse primer (Rev).

### Urine amino acid content

Six mice of each genotype were kept in metabolic cages to collect 24h urine samples and amino acids content was analyzed. Urine samples were diluted with 0.3M LiOH to solubilize possible cystine crystals and NorLeucine (2000μM) was added as an internal control. Urine samples were dried and resuspended with lithium citrate Loading Buffer pH 2.20 (Biochrom Ltd.). Quantitative analysis of amino acids was performed by chromatographic separation by cation‐exchange chromatography followed by post‐column derivatization with ninhydrin and UV detection in an amino acid analyzer (Biochrom Ltd.). The amount of **a**mino acids were identified according to the retention times of corresponding standards. Amount of amino acids were calculated by internal standard NorLeucine and the standards, and normalized by the volume of the analyzed sample.

### Calculi detection

Cystine calculi in the urinary system were detected by X‐ray radiography (at 28kV, 16 mA∙s) using a Senima HF mammography apparatus [19]. Mice were under isofluorane anesthesia. Mice of each genotype were X-rayed at 2, 5 and 8 months of age.

### Eosin/Hematoxylin staining

2 random paraffinized kidney tissue sections of 6 mice from each genotype were used for histopathology analysis. Slides were stained with 0.1% Mayers Hematoxylin and 0.5% Eosin solutions. Glomerular, tubular, interstitial and vascular lesions were scored by a nephropathologist based on a 4-tier system (0: absent, 1: mild, 2: moderate, 3: severe) similar to the one normally used to ascertain human kidney allograft lesions.

### Apical membranes (brush‐borders) Western blot

Apical membranes from 5 single mouse kidneys from each genotype mice were extracted following published protocol [20]. In order to detect the presence of the heterodimer [b^0,+^AT (37kDa) + rBAT(90kDa)] (120kDa), all SDS-polyacrylamide gels were run in the absence of reducing agents such as DTT or **β-**Mercaptoethanol to avoid heterodimer’s disruption. Both rBAT and b^0,+^AT were rabbit polyclonal antibodies against mouse proteins produced in our laboratory [17]. Antibodies were hybridized overnight at 4°C using 1/1000 dilution in 1% of skimmed milk. Protein detection was done by ECL (GE Healthcare) reaction and film exposure (FujiFilm). Densitometric analysis was done using Image J software. Statistical analysis performed was the unpaired Student’s t-test using GraphPad Prism 4.0 software.

## Supporting Information

S1 Fig Graph of body weight. **A.** Body weight at different months of 8–10 single homozygous mice (*Slc7a9* ^‐/‐^ *Slc3a1* ^*+/+*^ and *Slc7a9* ^+/+^ *Slc3a1* ^‐/‐^, respectively) and 7 double mutants (*Slc7a9* ^‐/‐^ *Slc3a1* ^*-/-*^). **B**. Graph of body weight at different months of 10 wild type mice and 12 double heterozygous.
(TIF)
