# Supplementary figures and images for: Digenic Inheritance in Cystinuria Mouse Model
Source: PLoS One. 2015 Sep 11;10(9):e0137277. doi: 10.1371/journal.pone.0137277 (PMC4567282; doi:10.1371/journal.pone.0137277)

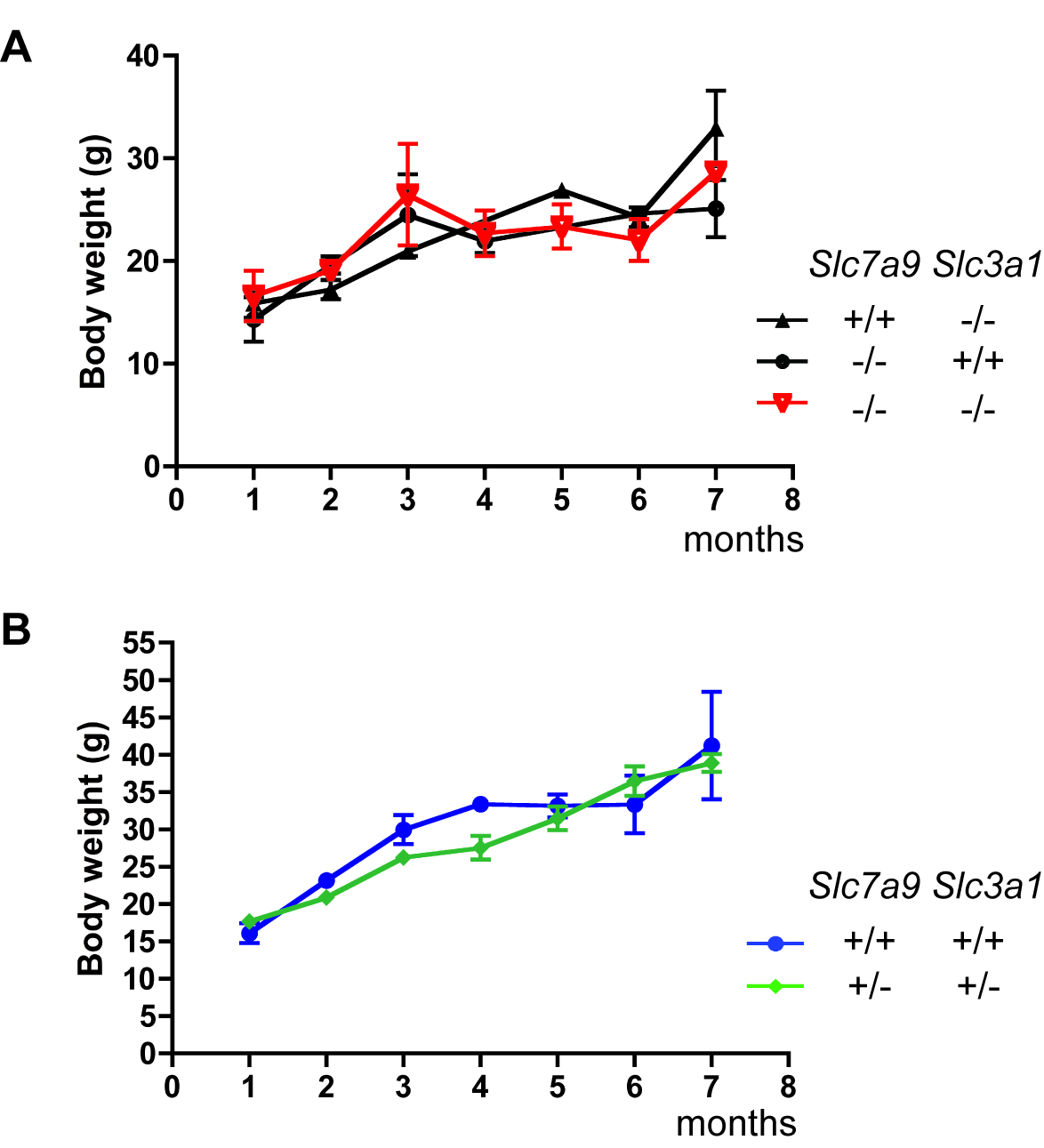

Supplement: S1 Fig — A. Body weight at different months of 8–10 single homozygous mice (Slc7a9 ‐/‐ Slc3a1 +/+ and Slc7a9 +/+ Slc3a1 ‐/‐, respectively) and 7 double mutants (Slc7a9 ‐/‐ Slc3a1 -/-). B. Graph of body weight at different months of 10 wild type mice and 12 double heterozygous. (TIF) [file pone.0137277.s001.tif]
